# Probing Proteostatic Stress in Degenerating Photoreceptors Using Two Complementary *In Vivo* Reporters of Proteasomal Activity

**DOI:** 10.1523/ENEURO.0428-19.2019

**Published:** 2020-01-02

**Authors:** Paige M. Dexter, Ekaterina S. Lobanova, Stella Finkelstein, Vadim Y. Arshavsky

**Affiliations:** 1 The Department of Pharmacology and Cancer Biology, Duke University School of Medicine, Durham, NC 27710; 2 Albert Eye Research Institute, Duke University, Durham, NC 27710

**Keywords:** photoreceptor, proteasome, proteostasis, retinal degeneration

## Abstract

Inherited retinal degenerations originate from mutations in >300 genes, many of which cause the production of misfolded mutant photoreceptor proteins that are ultimately degraded by the ubiquitin-proteasome system (UPS). It was previously shown that rod photoreceptors in multiple mouse models of retinal degeneration suffer from proteostatic stress consisting of an insufficient cellular capacity for degrading UPS substrates. In this study, we focused on a specific UPS component required for the degradation of a subset of proteasome targets: the substrate-processing complex formed by the AAA+ ATPase P97/VCP and associated cofactors. To assess whether P97 capacity may be insufficient in degenerating rods, we employed two complementary *in vivo* proteasomal activity reporters whose degradation is either P97-dependent or P97-independent. Retinal accumulation of each reporter was measured in two models of retinal degeneration: the transducin *γ*-subunit knock-out (*Gγ_1_^-/-^*) and P23H rhodopsin knock-in (P23H) mice. Strikingly, the patterns of reporter accumulation differed between these models, indicating that the proteostatic stress observed in *Gγ_1_^-/-^* and P23H rods likely originates from different pathobiological mechanisms, in which UPS substrate degradation may or may not be limited by P97-dependent substrate processing. Further, we assessed whether P97 overexpression could ameliorate pathology in *Gγ_1_^-/-^* mice, in which proteostatic stress appears to result from P97 insufficiency. However, despite P97 overexpression being aphenotypic in other tissues, the ∼2.4-fold increase in retinal P97 content was toxic to rods, which complicated the interpretation of the observed phenotype. Our results highlight the complexity of pathophysiological mechanisms related to degrading misfolded proteins in mutant photoreceptors.

## Significance Statement

Many mutations linked to inherited retinal degenerations cause the production of misfolded photoreceptor proteins, which overwhelms the capacity of the ubiquitin-proteasome system (UPS) in affected photoreceptors and causes proteostatic stress. This stress has been shown to be a major factor contributing to photoreceptor cell death in multiple mouse models of retinal degeneration. Here, we show that proteostatic stress in two common retinal degeneration models results from insufficient capacities of distinct components of the UPS. These results highlight the complexity of pathophysiological mechanisms related to degrading misfolded proteins in mutant photoreceptors, which must be accounted for in the development of effective strategies to ameliorate these blinding conditions.

## Introduction

Inherited retinal degenerations affect two to four million people worldwide. These conditions originate from mutations in >300 genes (https://sph.uth.edu/retnet/), many of which cause protein misfolding in affected photoreceptors. Previous work in multiple mouse models of retinal degeneration has indicated that the requirement to process large amounts of these misfolded proteins causes proteostatic stress, consisting of an insufficient capacity of the ubiquitin-proteasome system (UPS) to degrade proteasome substrates (Lobanova et al., 2013; Liu et al., 2014; Dexter et al., 2018). Conversely, genetic manipulation reducing the proteolytic capacity of proteasomes evokes photoreceptor death in otherwise normal retinas (Ando et al., 2014). It is also argued that proteasomal rather than lysosomal protein degradation is critical for photoreceptor survival in at least one retinal degeneration mouse model (Yao et al., 2018; Qiu et al., 2019). Importantly, ameliorating proteostatic stress by increasing proteasomal activity delayed photoreceptor death in mouse models of retinal degeneration (Lobanova et al., 2018). Despite this progress, the mechanistic understanding of the connection between insufficient UPS function and photoreceptor degeneration remains limited.

Protein degradation by the UPS is typically initiated by polyubiquitination of a target protein substrate, which enables the proteasome to recognize and degrade this protein (Finley, 2009). However, before being degraded by the proteasome, many proteins undergo a preliminary processing step performed by complexes formed by a homo-hexamer of the AAA+ ATPase P97 (also known as VCP) and multiple cofactors. P97 complexes are expressed in all cells and participate in a wide range of functions, including protein degradation, membrane fusion, vesicular trafficking, Golgi formation, autophagy, and DNA repair (Meyer and Weihl, 2014; Stach and Freemont, 2017). This versatility originates from the ability of P97 hexamers to associate with various combinations of over 40 interacting cofactors, which confer functional specificity to P97 complexes by regulating substrate interaction and subcellular localization (Buchberger et al., 2015; Hänzelmann and Schindelin, 2017).

In the context of the UPS, P97 complexes perform several well-established tasks that facilitate the degradation of substrates otherwise inaccessible to proteasomes. First, these complexes participate in extracting unfolded proteins from the endoplasmic reticulum (ER) during ER-associated protein degradation (Bays et al., 2001; Ye et al., 2001; Braun et al., 2002; Jarosch et al., 2002; Rabinovich et al., 2002). Second, they perform partial unfolding of certain polyubiquitinated protein substrates before proteasome entry (Wójcik et al., 2006; Beskow et al., 2009; Blythe et al., 2017; for review, see van den Boom and Meyer, 2018). This may be particularly important for generating the unstructured regions required for initiating protein degradation by proteasomes (Olszewski et al., 2019). Third, P97 complexes can edit polyubiquitin chains on proteins destined for degradation to optimize their proteasomal recognition (Koegl et al., 1999). It has also been hypothesized that P97 complexes may directly bind and feed substrates into the 20S proteolytic core of the proteasome, thereby replacing the 19S caps normally performing this function; however, it remains unclear whether such interaction occurs in eukaryotic cells (Barthelme and Sauer, 2012, 2013).

In this study, we investigated whether insufficient UPS function observed in degenerating rod photoreceptors could result, at least in part, from an insufficient cellular capacity for substrate processing by P97 complexes. We examined two complementary models of progressive retinal degeneration: the knock-out mouse lacking the *γ*-subunit of the rod G protein transducin (the *Gγ_1_^-/-^* mouse; Lobanova et al., 2008) and the knock-in mouse bearing a single copy of the P23H mutation in rhodopsin (the P23H mouse; Sakami et al., 2011). Rods of both models were previously documented to suffer from proteostatic stress. In *Gγ_1_^-/-^* mice, this stress results from the production of transducin’s β-subunit (Gβ_1_), which is unable to fold in the absence of G*γ*_1_ (Lobanova et al., 2013). In P23H mice, mutant rhodopsin misfolds in the ER, causing proteostatic stress (Lobanova et al., 2018) and eventually initiating the unfolded protein response and photoreceptor cell death (Chiang et al., 2015). Importantly, in both models, the associated misfolded protein undergoes highly efficient intracellular degradation and does not accumulate in the affected rods (Lobanova et al., 2008; Sakami et al., 2011).

Previously, proteostatic stress in these mouse models was demonstrated using the proteasomal activity reporter Ub^G76V^-GFP (Dantuma et al., 2000), which accumulated robustly in degenerating *Gγ_1_^-/-^* and P23H rods (Lobanova et al., 2013, 2018; Dexter et al., 2018). Because Ub^G76V^-GFP degradation by proteasomes requires its partial unfolding by P97 complexes (Wójcik et al., 2006; Beskow et al., 2009; Blythe et al., 2017), Ub^G76V^-GFP accumulation in these cells could indicate insufficient capacities of either of these UPS components. Thus, to assess whether the proteostatic stress observed in *Gγ_1_^-/-^* and P23H rods may be caused by insufficient P97-dependent substrate processing, we additionally monitored the accumulation of an alternative, P97-independent proteasomal activity reporter, oxygen-dependent degradation domain-Luciferase (ODDLuc; Safran et al., 2006).

Our analysis revealed a striking difference in the patterns of Ub^G76V^-GFP and ODDLuc reporter accumulation in *Gγ_1_^-/-^* and P23H retinas. While retinas of *Gγ_1_^-/-^* mice exhibited efficient clearance of the P97-independent ODDLuc reporter and accumulation of the P97-dependent Ub^G76V^-GFP reporter, both reporters accumulated in the retinas of P23H mice. These data suggest that the proteostatic stress experienced by these mice likely originates from different pathophysiological mechanisms in which protein degradation by the UPS may or may not be limited by the cellular capacity for P97-dependent substrate processing. We also assessed whether P97 overexpression could ameliorate pathology in *Gγ_1_^-/-^* retinas, in which proteostatic stress appears to result from P97 insufficiency. However, P97 overexpression was toxic to photoreceptors, which greatly complicated the interpretation of the observed phenotype.

Our results highlight the complexity of pathophysiological mechanisms related to degrading misfolded proteins in mutant rods. This complexity must be accounted for in the development of effective strategies to ameliorate these blinding conditions.

## Materials and Methods

### Animals

Mouse care and experiments were performed in accordance with procedures approved by the Institutional Animal Care and Use Committee of Duke University. The Deltagen Gγ_1_ knock-out (*Gγ_1_^-/-^*) mouse was licensed from Deltagen, Inc. (Target ID 408) and was previously characterized in (Lobanova et al., 2008, 2013). In this mouse, regions of the Gγ_1_ coding sequence (amino acids 17–44 and intron 2) were replaced with a 6.9 kb IRES-lacZ reporter and neomycin resistance cassette. P23H rhodopsin mutant knock-in (P23H) mice, characterized in (Sakami et al., 2011), were purchased from The Jackson Laboratory (stock #017628). Mice heterozygous for the P23H rhodopsin mutation were used for experiments.

Transgenic mice heterozygously expressing the Ub^G76V^-GFP reporter are described in (Lindsten et al., 2003). Transgenic mice expressing the ODDLuc reporter, characterized in (Safran et al., 2006), were obtained from The Jackson Laboratory (stock #006206). Both reporters were bred into the *Gγ_1_^-/-^* and P23H strains, and reporter experiments were performed using mutant mice heterozygously expressing either reporter. Breeding these mice also required breeding out the Rd1 mutation that causes severe retinal degeneration from ODDLuc mice. The transgenic mouse overexpressing wild-type (WT) human P97/VCP (the P97oe mouse) was previously characterized in (Custer et al., 2010) and was provided by J. Paul Taylor (St. Jude Children’s Research Hospital). Single copies of the Ub^G76V^-GFP and P97oe transgenes were bred into the *Gγ_1_^-/-^* line for Ub^G76V^-GFP quantification from retinal lysates. Littermates lacking Ub^G76V^-GFP expression were used for morphologic analyses.

Transgenic mice were maintained through heterozygous breeding with C57BL/6J WT mice from The Jackson Laboratory (stock #000664) and tested for the lack of Rd1 and Rd8 mutations. The WT control mice used in Figure 3*B* were littermates of experimental mice. Non-littermate C57BL/6J WT mice were used in other experiments. Mice of either sex were used for all experiments.

### Western blotting

For Western blot analysis of P97 and Ub^G76V^-GFP protein levels in retinal lysates, two mouse retinas per sample were solubilized in 150 μl of 1% Triton X-100 in PBS. Lysates were centrifuged at 16,850 × *g* for 15 min at 4°C, and supernatants were collected. Total protein concentration was measured using the DC Protein Assay kit (Bio-Rad), and samples were diluted with SDS-PAGE sample buffer. Proteins were separated by SDS-PAGE.

### Antibodies

Mouse anti-P97 antibody (ab11433, 1:2000) was from Abcam, rabbit anti-Hsc70 antibody (AD1-SPA-819, 1:10,000) was from Enzo Life Sciences, and mouse anti-eGFP antibody (632381, 1:5000) was from Clontech. Secondary goat or donkey antibodies conjugated with Alexa Fluor 680 and 800 (1:10,000) were from Invitrogen. Protein bands were visualized and quantified using the Odyssey Infrared Imaging System (LI-COR Biosciences).

### Luciferase activity assay

For quantitative analysis of ODDLuc reporter levels, luciferase activity was measured in retinal lysates of three- to four-week-old WT, *Gγ_1_^-/-^*, and P23H mice expressing a single copy of ODDLuc. As a positive control for reporter accumulation, ODDLuc heterozygotes were administered 1 μg Roxadustat/FG-4592 (Selleckchem) by intraocular injection, and their retinas were collected 4 h after injection. For each sample, two retinas were lysed in 150 μl ice-cold Glo Lysis buffer (Promega) and homogenized by pulse sonication. Retinal extracts were centrifuged at 500 × *g* for 10 min at 4°C, and the supernatants were centrifuged at 100,000 × *g* for 1 h at 4°C. The supernatants containing the soluble protein fraction were collected for use in the luciferase activity assay and diluted to 1 μg/μl with Glo Lysis buffer.

The luciferase activity assay was performed using the Bright-Glo Luciferase Assay System (Promega) according to the manufacturer’s protocol. Samples and reagents were thawed to room temperature, and 100 μl of retinal lysate (containing 100 μg of total protein) and 100 μl of Bright-Glo reagent were added to each well of a white, flat-bottom 96-well plate and mixed by gentle pipetting. Luminescence was immediately measured at all emission wavelengths 1×/min for 20 min using a SpectraMax M5 luminometer (Molecular Devices) with the following settings: Automix Once; measurement delay 2 s; measurement read 10 s; one read per well. Data were analyzed using the SoftMax Pro 4.8 software to plot relative luminescence units (RLU) as a function of time. For each sample, RLU at time 0 was determined by identifying the y-intercept of the line-of-best-fit for the RLU values measured. Data were normalized by dividing experimental RLU values by the averaged RLU values obtained from ODDLuc heterozygote control lysates included in each plate.

### Histology and microscopy

Photoreceptor cell loss was evaluated in semi-thin plastic-embedded retinal cross-sections (0.5 μm thick) obtained from mice at three and six months of age. The retinal cross-sections were prepared as follows: eyes were enucleated from mice and fixed for 1 h in freshly prepared 2% formaldehyde with 2.5% glutaraldehyde in 0.1 M cacodylate buffer containing 2.5 mM CaCl_2_ (pH 7.4). The eye globe was then hemisected along the vertical meridian and allowed to fix overnight in the same buffer. The eyecup was rinsed with excess 0.1 M cacodylate buffer (pH 7.4), placed into 2% osmium tetroxide for 1.5 h, and rinsed twice with water. The eye cup was gradually dehydrated in an increasing ethanol series (25–100%) and embedded in Epon; 0.5-μm cross-sections were obtained and stained with toluidine blue for light microscopy. Tiled images of whole retina cross-sections were obtained using the Nikon A1R confocal microscope and aligned and stitched using the NIS-Elements AR software. The number of photoreceptor nuclei in representative segments of outer nuclear layer was counted as a quantitative measure of surviving photoreceptors. Nuclear counts were performed in eight 100-μm segments located at 500-μm steps from the optic nerve head.

### Statistical analysis

All data are presented as the mean ± SD. Statistical analyses were performed with GraphPad Prism 7.04. *P* values are shown in the Statistical Table (Table 1). Results were considered statistically significant when *p* < 0.05. Data structure: For all comparisons, N was too small to determine whether data were normally distributed.

**Table 1.** Statistical table

Line	Figure	Comparison	Type of test	*p* value
a	Fig. 2*A*	GFP level in *Gγ_1_^-/-^* vs WT	Mann–Whitney	0.0007
b	Fig. 2*A*	GFP level in P23H vs WT	Mann–Whitney	0.0007
c	Fig. 2*A*	GFP level in *Gγ_1_^-/-^* vs P23H	Mann–Whitney	0.0022
d	Fig. 2*B*	Luc activity in Rox WT vs untreated	Mann–Whitney	0.0001
e	Fig. 2*B*	Luc activity in P23H vs WT	Mann–Whitney	0.0002
f	Fig. 2*B*	Luc activity in *Gγ_1_^-/-^*vs WT	Mann–Whitney	<0.0001
g	Fig. 2*B*	Luc activity in *Gγ_1_^-/-^* vs P23H	Mann–Whitney	0.0016
h	Fig. 3*A*	P97 level in *Gγ_1_^-/-^* vs WT	Mann–Whitney	0.8182
i	Fig. 3*A*	P97 level in P23H vs WT	Mann–Whitney	0.7056
j	Fig. 3*B*	P97 level in P97oe vs WT	Mann–Whitney	0.0079
k	Fig. 3*D*	Nuclei in P97oe vs WT at three months	Mann–Whitney	0.0357
l	Fig. 4*B*	Nuclei in *Gγ_1_^-/-^*/P97oe vs *Gγ_1_^-/-^* at three months	Mann–Whitney	0.2
m	Fig. 4*E*	Nuclei in *Gγ_1_^-/-^*/P97oe vs *Gγ_1_^-/-^* at six months	Mann–Whitney	0.0095
n	Fig. 5	GFP level in *Gγ_1_^-/-^*/P97oe vs *Gγ_1_^-/-^*	Mann–Whitney	0.0079

## Results

### Comparison of Ub^G76V^-GFP and ODDLuc accumulation in *Gγ_1_^-/-^* and P23H retinas

**Figure 1.**
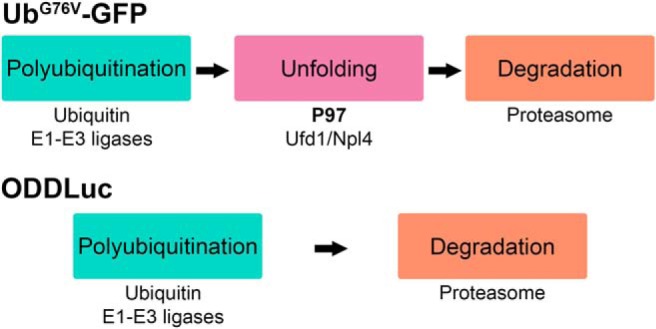
The *in vivo* proteasomal activity reporters Ub^G76V^-GFP and ODDLuc are degraded through P97-dependent and P97-independent mechanisms. Top, Ub^G76V^-GFP is a fusion protein consisting of GFP fused to a non-cleavable molecule of ubiquitin, which acts as a constitutively active degradation signal. Ub^G76V^-GFP is degraded by proteasomes following its polyubiquitination and partial unfolding by the P97-Ufd1-Npl4 complex. Bottom, ODDLuc is a fusion protein consisting of firefly luciferase fused to an alternative degradation signal, the ODD of HIF1α. This reporter is polyubiquitinated and degraded by proteasomes without P97 processing. Conditions of inhibited, impaired, or insufficient UPS function result in the intracellular accumulation of either reporter.

We first assessed P97-dependent UPS substrate processing in *Gγ_1_^-/-^* and P23H mice by monitoring the retinal accumulation of two *in vivo* proteasomal activity reporters whose degradation is either P97-dependent or P97-independent (Fig. 1). The first reporter, Ub^G76V^-GFP (Dantuma et al., 2000), is degraded by proteasomes following its partial unfolding by the P97-Ufd1-Npl4 substrate-processing complex (Wójcik et al., 2006; Beskow et al., 2009; Blythe et al., 2017). The second reporter, ODDLuc (Safran et al., 2006), is efficiently degraded by proteasomes without P97 involvement (Chou and Deshaies, 2011). The P97 independence of ODDLuc was previously established using a cell line expressing both Ub^G76V^-GFP and ODDLuc. Treatment of these cells with proteasome inhibitor blocked the degradation of both reporters. On the other hand, P97 inhibition or its siRNA knock-down blocked the degradation of Ub^G76V^-GFP but not ODDLuc (Chou and Deshaies, 2011).

**Figure 2.**
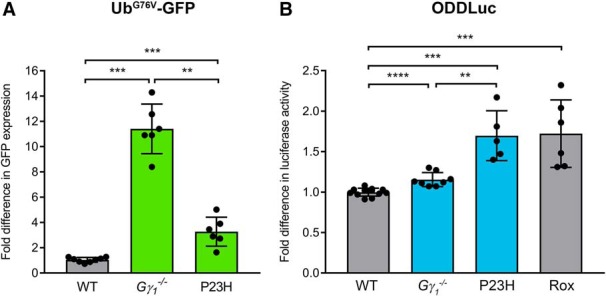
Comparative analysis of proteasomal activity reporter accumulation in *Gγ_1_^-/-^* and P23H retinas. ***A***, The Ub^G76V^-GFP reporter was detected in retinal lysates (25 μg total protein/lane) of three- to four-week-old *Gγ_1_^-/-^* or P23H mice by Western blotting with an anti-GFP antibody; the densities of the Ub^G76V^-GFP bands were normalized to the Hsc70 loading control. The number of mice analyzed was the following: WT, 8; *Gγ_1_^-/-^*, 6; P23H, 6. Each dot represents a single data point. ***B***, The ODDLuc reporter was detected in retinal lysates (100 μg total protein/reaction) from three- to four-week-old *Gγ_1_^-/-^* or P23H mice using the Bright-Glo Luciferase Assay system. Retinal lysates from ODDLuc mice treated with the HIF prolyl hydroxylase inhibitor Roxadustat (Rox) were used as a positive control for reporter accumulation. The number of mice analyzed was the following: WT, 12; *Gγ_1_^-/-^*, 8; P23H, 5; Rox, 6. Each dot represents a single data point. Data are shown as the mean ± SD. **p < 0.01, ***p < 0.001, ****p < 0.0001.

We bred *Gγ_1_^-/-^* and P23H mice with the mice ubiquitously expressing either Ub^G76V^-GFP (Lindsten et al., 2003) or ODDLuc (Safran et al., 2006) and assessed the accumulation of each reporter in the retinas of three- to four-week-old mice, in which photoreceptor degeneration has just begun. As reported previously (Lobanova et al., 2013, 2018; Dexter et al., 2018), Ub^G76V^-GFP accumulated in retinas of both *Gγ_1_^-/-^* and P23H mice (Fig. 2*A*). Retinal Ub^G76V^-GFP content increased 11.5 ± 2.0-fold (*p* = 0.0007) in *Gγ_1_^-/-^* and 3.3 ± 1.2-fold (*p* = 0.0007) in P23H mice relative to WT controls.^a,b^

The accumulation of ODDLuc in retinal lysates of *Gγ_1_^-/-^* and P23H mice was monitored using the luciferase activity assay. As a positive control for ODDLuc accumulation, we injected the HIF prolyl hydroxylase inhibitor Roxadustat/FG-4592 in the vitreous of ODDLuc heterozygotes and collected their retinas 4 h after injection. This treatment caused an increase in ODDLuc accumulation of 1.7 ± 0.4-fold (*p* = 0.0001; Fig. 2*B*),^d^ which we considered as a benchmark of this reporter’s sensitivity. Notably, this signal was smaller than the signal produced by Ub^G76V^-GFP, yet sufficiently significant for quantitative analysis.

**Figure 3.**
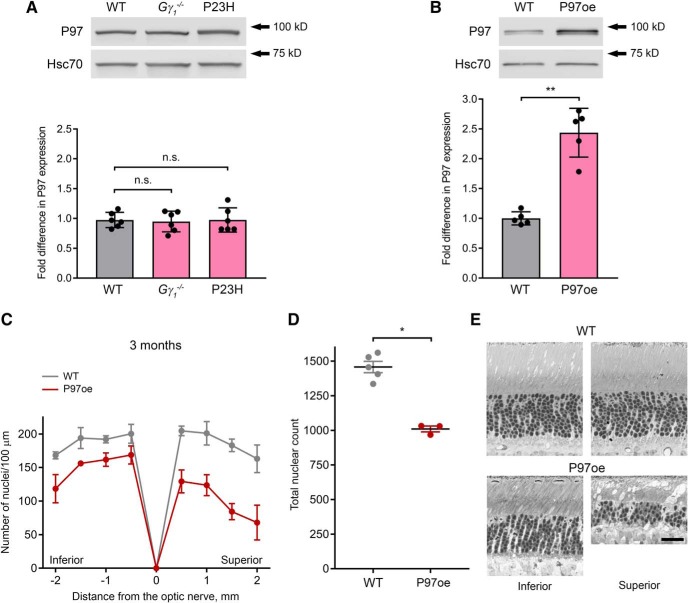
Overexpression of P97 affects photoreceptor survival. ***A***, P97 protein level was detected in retinal lysates (2 μg total protein/lane) of one-month-old *Gγ_1_^-/-^* or P23H mice by Western blotting with an anti-P97 antibody; the densities of the P97 bands were normalized to the Hsc70 loading control. The number of mice analyzed was the following: WT, 6; *Gγ_1_^-/-^*, 6; P23H, 6. Each dot represents a single data point. ***B***, P97 protein level was detected in retinal lysates (2 μg total protein/lane) from one-month-old WT and P97-overexpressing (P97oe) mice as in ***A***; Hsc70 was used as a loading control. The number of mice analyzed was the following: WT, 5; P97oe, 5. Each dot represents a single data point. ***C***, Spider diagrams representing the number of photoreceptor nuclei in 100-μm segments of the inferior and superior retina at various distances from the optic nerve head. The number of mice analyzed was the following: WT, 5; P97oe, 3. Mice were three months old. ***D***, The total number of nuclei in all eight 100-μm retinal segments represented in spider diagrams of panel ***C***. Each dot represents a single data point. ***E***, Representative images of inferior and superior retina cross-sections from WT and P97oe at the 1-mm distance from the optic nerve head. Mice were three months old. Scale bar = 25 μm. Data are shown as mean ± SD. n.s. indicates *p* > 0.05. *p < 0.05, **p < 0.01.

We next analyzed ODDLuc accumulation in the retinas of mutant mice (Fig. 2*B*). ODDLuc accumulation in P23H retinas increased 1.7 ± 0.3-fold (*p* = 0.0002), reaching the same level as observed in the Roxadustat control.^e^ On the other hand, ODDLuc accumulation in *Gγ_1_^-/-^* retinas increased by only 1.2 ± 0.1-fold (*p* < 0.0001).^f^ Notably, this pattern was qualitatively opposite to the pattern of Ub^G76V^-GFP accumulation, which was higher in *Gγ_1_^-/-^* retinas. This difference suggests that, though both *Gγ_1_^-/-^* and P23H rods experience proteostatic stress, this condition likely results from insufficient capacities of different UPS components. The relatively high level of ODDLuc accumulation in P23H retinas suggests that UPS function in their rods is limited by an insufficient cellular capacity for proteasomal degradation. On the other hand, the combination of a high level of Ub^G76V^-GFP accumulation and a low level of ODDLuc accumulation in *Gγ_1_^-/-^* retinas suggests that UPS function in this model may be limited by P97-dependent substrate processing. The latter could not be explained merely by a reduction in the expression of P97, as P97 expression levels were essentially indistinguishable from WT and identical in the retinas of both mouse models (Fig. 3*A*).^h,i^

### Characterization of the retinal morphology of the P97-overexpressing mouse

We next followed up on our observation that proteostatic stress in *Gγ_1_^-/-^* retinas may result from insufficient P97-dependent substrate processing. Currently, there are no available strategies for increasing the substrate-processing capacity of P97 complexes in an *in vivo* context. We attempted to accomplish this through the most straightforward strategy of overexpressing P97 itself. For this purpose, we obtained the transgenic mouse line ubiquitously overexpressing WT human P97 (the P97oe mouse), described in Custer et al. (2010). The P97oe mouse was characterized as phenotypically normal, with no change in development, weight, or survival compared to WT mice. However, the retinal phenotype of this mouse was not assessed.

We first determined the P97 content in P97oe retinas (Fig. 3*B*). Quantitative Western blotting of retinal lysates from one-month-old mice indicated a robust, 2.4 ± 0.4-fold increase in their P97 content (*p* = 0.0079).^j^ This overexpression level was essentially the same as previously reported for the brain (Custer et al., 2010). We next investigated whether this increase in P97 expression affects photoreceptor health by analyzing the retinal morphology of P97oe mice in thin plastic sections from three-month-old mice (Fig. 3*C–E*). To quantify the effect of P97 overexpression on photoreceptor survival, we counted the number of photoreceptor nuclei in 100-μm retinal segments at different distances from the optic nerve and plotted the results as a spider diagram (Fig. 3*C*). We found that P97oe retinas exhibited marked photoreceptor loss at all analyzed regions of the retina, with the total nuclear count obtained from these regions decreasing by ∼31% compared to WT controls (from 1458 ± 91 nuclei in WT to 1010 ± 36 in P97oe, *p* = 0.0357; Fig. 3*D*).^k^ We also analyzed retinas from P97oe mice at six months of age and observed ∼42% photoreceptor loss compared to WT (data not shown).

These data indicate that, although P97oe mice were not previously reported to display any obvious neurologic abnormalities (Custer et al., 2010), the ∼2-fold increase in retinal P97 content in these mice is toxic to rod photoreceptors and causes significant cell death before three months of age. Additionally, we observed that that the toxic effect of P97 overexpression was more pronounced in the superior retina than in the inferior retina (Fig. 3*C*,*E*).

### Photoreceptor degeneration in *Gγ_1_^-/-^* mice overexpressing P97

**Figure 4.**
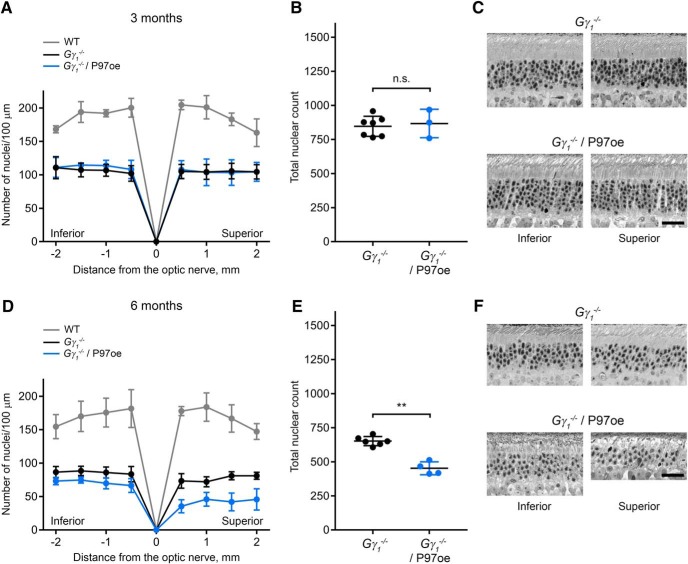
Overexpression of P97 affects photoreceptor survival in *Gγ_1_^-/-^* mice. ***A–F***, Morphology of *Gγ_1_^-/-^* retinas with or without P97 overexpression at three (***A–C***) or six (***D–F***) months of age. ***A***, ***D***, Spider diagrams representing the number of photoreceptor nuclei in 100-μm segments of the inferior and superior retina at various distances from the optic nerve head. The number of mice analyzed at three months of age was the following: WT, 3; *Gγ_1_^-/-^*, 7; *Gγ_1_^-/-^*/P97oe, 3. The number of mice analyzed at six months of age was the following: WT, 4; *Gγ_1_^-/-^*, 6; *Gγ_1_^-/-^*/P97oe, 4. ***B***, ***E***, The total number of nuclei in all eight 100-μm retinal segments represented in panels ***A***, ***D***. ***C***, ***F***, Representative images of inferior and superior retina cross-sections at the 1-mm distance from the optic nerve head. Scale bar = 25 μm. Data are shown as mean ± SD. n.s. indicates *p* > 0.05. **p < 0.01.

Despite observing some P97 overexpression toxicity, we nonetheless decided to evaluate whether P97 overexpression may improve photoreceptor survival in *Gγ_1_^-/-^*, which apparently suffers from P97 insufficiency. We bred the *Gγ_1_^-/-^* mouse with the P97oe mouse and assessed photoreceptor survival in the resulting line at three and six months of age (Fig. 4). At three months of age, *Gγ_1_^-/-^* and *Gγ_1_^-/-^*/P97oe retinas exhibited a nearly identical degree of photoreceptor loss (Fig. 4*A–C*).^l^ By six months of age, *Gγ_1_^-/-^*/P97oe retinas exhibited a slight increase in photoreceptor loss compared to *Gγ_1_^-/-^* retinas (453 ± 47 in *Gγ_1_^-/-^*/P97oe vs 652 ± 34 nuclei in *Gγ_1_^-/-^*, *p* = 0.0095; Fig. 4*D–F*).^m^ Once again, the superior retina was affected to a larger degree than the inferior retina. Thus, P97 overexpression only exacerbated the deleterious effects of Gγ_1_ knock-out.

### Ub^G76V^-GFP accumulation in *Gγ_1_^-/-^* retinas overexpressing P97

**Figure 5.**
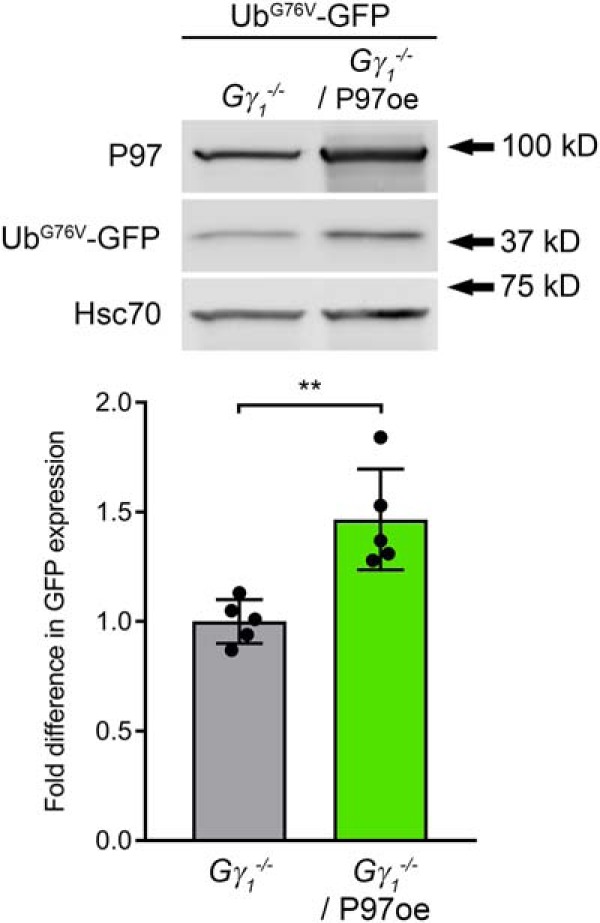
Overexpression of P97 causes increased accumulation of the Ub^G76V^-GFP reporter. The Ub^G76V^-GFP reporter was detected in retinal lysates (25 μg total protein/lane) of one-month-old *Gγ_1_^-/-^* and *Gγ_1_^-/-^*/P97oe mice by Western blotting with an anti-GFP antibody; the densities of the Ub^G76V^-GFP bands were normalized to the Hsc70 loading control. The number of mice analyzed was the following: *Gγ_1_^-/-^*, 5; *Gγ_1_^-/-^*/P97oe, 5. Each dot represents a single data point. Data are shown as the mean ± SD. **p < 0.01.

Finally, we investigated whether P97 overexpression actually facilitated P97-dependent substrate processing in *Gγ_1_^-/-^* photoreceptors. To do this, we bred single copies of the P97 and Ub^G76V^-GFP transgenes into the *Gγ_1_^-/-^* line. Ub^G76V^-GFP reporter accumulation in the resulting mouse was quantified by Western blotting of retinal lysates at one month of age. Contrary to our expectations, retinas of *Gγ_1_^-/-^*/P97oe mice exhibited a slight but statistically significant (1.5 ± 0.2-fold; *p* = 0.0079) increase in Ub^G76V^-GFP accumulation compared to *Gγ_1_^-/-^* (Fig. 5).^n^ This result indicates that P97 overexpression increases the level of proteostatic stress already experienced by these mice.

## Discussion

Recent work has established that proteostatic stress, consisting of an insufficient capacity of the UPS to degrade proteasomal substrates, is a major contributing factor in multiple forms of inherited retinal degeneration (Lobanova et al., 2013; Liu et al., 2014; Dexter et al., 2018). In this study, we evaluated whether this proteostatic stress may originate from insufficient P97-dependent substrate processing, which is required for the proteasomal degradation of a large number of UPS targets. Our data indicate that this could be the case, although only for a subset of mutations associated with photoreceptor protein misfolding. Whereas insufficient UPS function in *Gγ_1_^-/-^* rods appears to result from insufficient P97-dependent substrate processing, proteasomal degradation itself appears to limit UPS function in P23H rods. These results argue that individual mutations must be considered when developing therapeutic interventions for reducing proteostatic stress in these blinding conditions.

Whereas the exact mechanistic explanation for the difference in the rate-limiting UPS components between these mutant mouse models remains to be addressed experimentally, we can offer the following hypothetical explanation consistent with our current results. Photoreceptors are known to constantly renew their light-sensitive membranes, which requires continuous biosynthesis of unusually large amounts of membrane proteins. This implies that they have a high capacity of the ER-associated degradation machinery, including P97 complexes, to perform quality control of proteins produced in the ER. One can speculate that this capacity is naturally so high that it is sufficient to process misfolded P23H rhodopsin molecules. However, these cells do not appear to have enough downstream proteasomes, ultimately leading to proteostatic stress in P23H mice. The cytoplasmic pool of P97 in photoreceptors may be more trivial and easily saturable by the large amounts of uncoupled Gβ_1_ produced in *Gγ_1_^-/-^* rods. Assuming that these cells lack the ability to redirect the ER-associated P97 pool to the cytoplasm, the proteostatic stress in *Gγ_1_^-/-^* photoreceptors arises from P97 insufficiency.

Interestingly, the most straightforward approach to overcoming insufficient P97-dependent substrate processing in *Gγ_1_^-/-^* photoreceptors, consisting of overexpressing this protein, was not productive. In fact, P97 overexpression caused progressive death of otherwise normal photoreceptors. Furthermore, P97 overexpression exacerbated proteostatic stress and accelerated photoreceptor loss in *Gγ_1_^-/-^* mice. The simplest explanation for these effects is that increased cellular content of P97 does not automatically lead to formation of additional functional P97 complexes and may instead impose its own stress on the UPS. However, recent studies of P97 mutations linked to human disease suggest a more complex explanation for the photoreceptor cell death we observe in P97oe mice.

Mutations in P97 can cause multisystem proteinopathy (MSP), a syndromic disorder affecting the brain, bone and muscle. Disease-associated mutations in P97 were recently shown to increase the rate of substrate unfolding by P97 complexes *in vitro* (Blythe et al., 2017, 2019), and expression of such ‘overactive’ P97 mutants in Drosophila and mice was highly toxic (Custer et al., 2010; Ritson et al., 2010; Zhang et al., 2017). These data raise a possibility that the toxicity observed in P97-overexpressing photoreceptors may result from a pathologic increase in P97-dependent substrate processing. While this possibility is not immediately supported by our finding that P97 overexpression increases Ub^G76V^-GFP accumulation in *Gγ_1_^-/-^* retinas, cell death in P97-overexpressing rods may arise from multiple contributing factors. For example, increased P97-dependent processing and degradation of regulatory proteins could inappropriately activate critical signaling pathways, such as NF-κB (Custer et al., 2010), leading to cellular dysfunction and cell death. Such perturbations could not be detected by merely monitoring cytosolic Ub^G76V^-GFP levels. Given the complexity of this system, understanding how to fine-tune P97-dependent substrate processing to alleviate the proteostatic stress caused by misfolded protein production will require a broader consideration of the entire P97-interacting network.

Curiously, photoreceptors are the only cells studied so far that are negatively affected by P97 overexpression. In the initial characterization of the P97oe mouse (Custer et al., 2010), these mice developed normally and displayed no abnormalities in weight or survival at all ages evaluated (up to 15 months of age). Kidney, liver, spleen, intestine, and gastrocnemius and quadriceps muscles of P97oe mice appeared histologically normal, despite confirmed transgene expression in these tissues. Brains harvested from P97oe mice, in which P97 expression was increased ∼2-fold, revealed no overt signs of degeneration by hematoxylin and eosin staining. P97oe mice also exhibited normal hind-limb clasping, a behavior which is disrupted in numerous mouse models of neurodegeneration and is a common indicator of central nervous system pathology (Reddy et al., 1999). Finally, tests of declarative memory and anxiety-associated behaviors also revealed no abnormalities in P97oe mice (Custer et al., 2010). Given the otherwise healthy phenotype of the P97oe mouse, the toxicity we observed in P97oe photoreceptors came as a surprise to us. However, what could be viewed as even more surprising is that only one cell type is negatively affected by 2- to 3-fold overexpression of P97, which is already one of the most abundantly-expressed proteins in the cell (Peters et al., 1990).

Finally, our new data make a mechanistic connection with a recent study in which we demonstrated that overexpression of the PA28α subunit of the 11S proteasome cap is therapeutic (Lobanova et al., 2018). In this study, we showed that PA28α overexpression increases proteasomal activity and delays photoreceptor cell loss in both *Gγ_1_^-/-^* and P23H mice. One observation that we could not immediately explain was that photoreceptor protection on proteasomal activation was much more pronounced in P23H mice. However, a plausible explanation can now be offered in light of our current findings. Our data suggest that the proteostatic stress in *Gγ_1_^-/-^* rods results from an insufficient cellular capacity for P97-dependent substrate processing, whereas P23H rods experience insufficient proteasomal degradation. Therefore, it is entirely expected that direct proteasomal activation would be more effective in P23H rods, whereas protection of *Gγ_1_^-/-^* rods would require increasing substrate processing by P97 complexes.

## Synthesis

Reviewing Editor: Kirill Martemyanov, The Scripps Research Institute Decisions are customarily a result of the Reviewing Editor and the peer reviewers coming together and discussing their recommendations until a consensus is reached. When revisions are invited, a fact-based synthesis statement explaining their decision and outlining what is needed to prepare a revision will be listed below. The following reviewer(s) agreed to reveal their identity: Sheila Baker, Gabriel Travis.

Upon discussion the reviewers agreed that the manuscript provides important advance to the field. It was felt that the data are of high quality and focus on protein misfolding in photoreceptor degeneration is an asset. The reviewers made a number of minor recommendations for further improving the manuscript and both agreed that no further experiments would be needed and the issues could be dealt with by textual changes. The unedited comments of the reviewers are provided below for reference.

Reviewer 1

This study builds on previous observations that some forms of retinal degeneration involve proteostatic stress through an imbalance in the amount of accumulated ubiquitylated proteins and the capacity of the proteasomes to degrade those substrates. The central focus in on the role of the unfoldase, P97. The amount of proteostatic stress as measured with a P97-dependent (Ub-G76V-GFP) and P97 independent (ODD-luc) transgenic line in two different models of retinal degeneration was measured. Interestingly, there was an inverse accumulation of these reporters when comparing the Gy KO and P23H strains, leading to the conclusion that protein degradation in Gy KO is limited by P97. To test this, the authors proceeded to cross an P97 over-expressing line to the Gy KO with the expectation that it would reduce retinal degeneration due to loss of Gy. The results are complicated by the finding of retinal degeneration in the P97 over-expressing line. There may be some protection evident in 3-month-old Gy KO/P97 oe animals but the authors are correctly cautious in their interpretation of the data. The paper is clearly written, and the data simply presented, making this a high-quality report. However, the interpretation of the ODD-luc data relies on the assumption that the ODDluc mice are reporting bypass of P97. The ODDluc mice are used in other contexts to report hypoxia and especially given the authors discovery that P97oe is toxic in retina even though that hadn't been reported in other tissues, a control showing bypass of P97 would greatly enhance the study. Additional comments follow:

1. The introduction and Figure 1 could be improved by providing additional context for P97. For example, is P97 the only ATPase that can partially unfold poly-uniquitylated proteins, does the substrate-processing complex interact directly with the core or caps of the proteasome, are there other known complexes that provide a similar function to the P97-containing complex, is there evidence of cell-type specificity for the adaptors used with P97?

2. Gy KO/P97oe (Fig 4) - it is interesting that combining the P97oe transgene with the Gy KO adds to the amount of degeneration at 6 months but not 3 months. Could this indicate a partial protection? Did the authors measure retinal degeneration in this line at 1 month, or conversely measure Ub-G76V-GFP accumulation (as in Fig 5) at 3 months?

3. How do the authors envision the different starting places for unfolded protein accumulation (ER for P23H, cytosol for Gbeta in absence of Gy) contributing to the requirement for P97?

4. Discussion (lines 295-298) - regarding the toxicity of P97oe, the authors could include commentary on the observation reported in the Blythe et al reference that a disease mutation has increased activity.

Minor clarifications

• Line 108 - by “expressing a 'single' copy” do the authors mean to say 'heterozygous'? The Ub-G76V-GFP transgenic line was originally described as having inserted into the genome in a head-to-tail array.

• The age of the animals used for histology is provided in the results, but it would be helpful to have that info included in the methods as it is for the luc assays

• When C57 animals are used as controls do they come from a separate colony or are they littermates of the various transgenes used in the study?

Reviewer #2

This manuscript studies the limiting components of the UPS in two mouse models of photoreceptor degeneration previously shown to be caused by proteostatic stress. The Gγ1-/- mouse lacks the gamma-subunit of rod transducin, while the P23H knockin mouse expresses a mutant form of rhodopsin associated with retinitis pigmentosa. In the Gγ1-/- mouse, stress arises from the normal Gβ subunit that does not fold due to loss of Gγ, while in the P23H mouse, the mutant rhodopsin itself causes stress. To discriminate between P97-dependent and P97-independent mechanisms, the authors looked for accumulation in retinal homogenates of the reporters, UbG76V-GFP and ODDLuc, respectively. UbG76V-GFP accumulated in both mutant mice, while ODDLuc mainly accumulated in the P23H mouse. These findings were interpreted to suggest that in Gγ1-/- retinas, UPS function is limited by P97 processing, while in P23H retinas UPS function is limited by insufficient proteasomal processing capacity. However, overexpression of P97 caused photoreceptor degeneration in wild type mice, and accelerated photoreceptor degeneration in Gγ1-/- mice. Finally, the authors tested whether P97 overexpression facilitated UPS in Gγ1-/- mice. Contrary to expectations, UbG76V-GFP increased in P97-overexpressing Gγ1-/- mice.

The study presented in this manuscript is well designed and controlled, the data are of high quality, and the conclusions are supported by the results. An important finding is that the biochemical mechanism of proteostatic stress in photoreceptors varies for different mutations. I would invite the authors to speculate about this in the Discussion. For example, might the difference relate to the fact that P23H-rhodopsin is an abnormal protein that folds improperly, while Gβ is a normal protein that should fold properly but is missing its Gγ partner in Gγ1-/- mice? The results of this study has broad relevance beyond vision science, hence eNeuro is an appropriate venue.
